# The Waiver of Patent Protections for COVID-19 Vaccines During the Ongoing Pandemic and the Conspiracy Theories: Lights and Shadows of an Issue on the Ground

**DOI:** 10.3389/fmed.2021.756623

**Published:** 2021-11-04

**Authors:** Matteo Nioi, Pietro Emanuele Napoli

**Affiliations:** ^1^Department of Medical Sciences and Public Health, Forensic Medicine Unit, University of Cagliari, Cagliari, Italy; ^2^Department of Surgical Sciences, Eye Clinic, University of Cagliari, Cagliari, Italy

**Keywords:** COVID-19 vaccines, COVID-19 vaccines patent, COVID-19 herd immunity, COVID-19 conspiracy theories, COVID-19 low-income countries, COVAX AMC, mRNA vaccine

## Introduction

Vaccines against the coronavirus disease 2019 (COVID-19) currently constitute the main hope for the fight against the ongoing pandemic. Mass immunization against the severe acute respiratory syndrome coronavirus 2 (SARS-Cov-2) should be considered as a global battle in which no country can be left behind, in particular the low-income countries. This last aspect may, in fact, favor the uncontrolled replication of the virus with the insurge of new variants. One of the possible solutions to limit this problem is the waiver of patent protections for COVID-19 vaccines.

On 2 October 2020, India and South Africa asked the World Trade Organization (WTO) to allow all countries to choose to neither grant nor enforce patents and other intellectual property (IP) related to COVID-19 drugs, vaccines, diagnostics and other technologies for the duration of the pandemic, until global herd immunity is achieved.

On 5 May 2021, the United States (US) administration announced the willingness to liberalize intellectual property concerning the COVID-19 vaccines (1, 2).

Although this issue is very interesting, its feasibility is particularly complex and intricate. Accordingly, there are discordant opinions among scholars and physicians on the real usefulness of patent liberalization (3–6). It has been suggested that an exception be made to the agreements establishing the World Trade Organization, in particular The Agreement on Trade Related Aspects of Intellectual Property Rights. This request would be based on the theory according to which the vaccines against the Severe Acute Respiratory Syndrome Coronavirus 2 (SARS-CoV-2) during a pandemic should be considered as a “common good”, similarly to water, or air. According to the Economic Theory of the Commons, which is supported by Elinor Ostrom, winner of the 2009 Nobel prize in Economics, the aforementioned terms should be considered in the Roman category of “res communes omnium”, meaning not appropriable and precluded from legal trade (7, 8).

From a practical point of view, the suspension of patent protections would limit the business models for the legitimate producers of COVID-19 vaccines. Among the potential strategies to encourage the waiver of patent protections it is possible to include: the direct donation of vaccines from the high-income countries, the reduction of the costs of the production lines, the shortening of the duration of patent protections, and a series of preferential patent waivers to countries that manufacture a large fraction of the global vaccines (e.g., India). One of the further possible solutions is represented by the solidarity by several governments or other agencies. This strategy is pursued, for example, by the program for *COVID-19 Vaccines Global Access* (COVAX). The latter is supported by Global Alliance for Vaccines and Immunization (GAVI), Coalition for Epidemic Preparedness Innovations (CEPI), and the World Health Organization (WHO) (9, 10).

## Positive Issues (Lights)

The thesis supported by Biden states that the waiver of patent protections could allow countries with fewer resources (e.g., Middle Africa) to reduce the price necessary to produce large quantities of vaccine (11, 12). Although vaccine prices are lower in low-income countries then in middle and high-income countries, and despite the productive effort of countries like India (considered 'the vaccine hub of the world'), their cost still does not make them accessible to all low-income countries (13) (Table 1).

**Table 1:** Prices of vaccines in different countries in US dollars ($).

**Vaccine price (single dose)**	**STK**	**AZ-C**	**AZ-V**	**J**	**SP**	**M**	**VPT**	**CN**	**CVC**	**CVX**
EU	10	-	2.19–3.5	8.5	-	18–25.5	9.3	23.15–18.9	-	-
USA	10	-	4	10	-	15	10.5	19.50	-	-
CH	10	-		-	29.75	-	-		29.75	-
IND	10	2.88–7.95	-	-	-	-	-		-	3.02–5.45
AU	19 (GH)	3	-	10	18.6 (SNG)	28.88 (BTW)	-	6.75	15 (BTW)	16 (BTW)
RSS	-	-		-	-	-	-		-	
BR	-	5		-	-	-	-	10	10.30	15
HU	19.9	-	3.16	-	36	-	-		-	
COVAX	-	3	-	-	-	-	-	-	-	-

*STK, Sputnik V; AZ-C, AstraZeneca Covishield; AZ-V, Varvexia; J, Ad26COV2.S Janssen; SP, BBIP-CorV Sinopharm; M, mRNA 1273 Moderna; VPT, VidPrevTyn; CN, Comirnaty, Pfizer/BioNTech; CVC, Coronavac; CVX, Covaxin; BTW, Botswana; SNG, Senegal; GH, Ghana; EU, European Union; USA, United States of America; CH, China; IND, India; AU, African Union; RSS, Russia; BR, Brazil; HU, Hungary; COVAX, COVAX AMC, COVID-19 Vaccines Advance Market Commitment*.

In this way, it would be possible to reach a larger portion of vaccinated population through autonomous production. Various politicians from multiple regions of the world have responded enthusiastically to this announcement.

In addition, the high- and middle-income countries would also benefit from this policy for various reasons. First, in this modern-day world that has developed increasingly blurred borders, a high likelihood that unvaccinated economic migrants arriving in high- and middle-income countries, for example in West Europe, would nullify the achievement of herd immunity exists.

The second, but no less important factor, regards the possibility that the waiver of patent protections for COVID-19 vaccines may be also extended to other potential molecules against SARS-CoV-2. This hypothesis has already been proposed during the request of India and South Africa in October 2020 which did not limit themselves to asking “to choose not to grant or enforce patents and other intellectual property (IP) related only to COVID-19 Vaccines” but also included COVID-19 drugs, diagnostics and other technologies and materials for the duration of the pandemic, until global herd immunity is achieved. Historically, the need for exceptional patent measures also occurred during a major HIV epidemic outbreak in South Africa in 1997. This event was important in outlining the principles contained in the 2001 Doha Declaration, which affirmed the need that developing countries, under certain conditions of health emergency, have the right to produce low-priced copies of medicines still protected by the patent (14, 15).

Last but not least point regards the discouragement of the spread of conspiracy theories developed in the West during this last year. In fact, almost all of the conspiracy theories identify an economic advantage or power for “Big Pharma” (intended as an hypothetical group of large pharmaceutical companies that operate for sinister purposes and against the public health) to the detriment of the health and safety of the individual citizen by damaging the rational principle of non-maleficence (see the chapter below entitled “Fight against conspiracy theories”).

### Fight Against Conspiracy Theories

Although it has been shown that a large percentage of supporters of the conspiracy theories (e.g., anti-vaxxers) suffer from phobias that prompt them to refuse the vaccine (e.g., fear of needles, fear of drugs, fear of doctors, etc.) (16), these groups have endorsed a series of arguments that have appeared alternatively totally-irrational or based on generic concepts taken to extremes without scientific evidences (thus becoming inductive hyperboles). Unfortunately, the crisis due to the current pandemic does not allow time to address and treat all these phobias (e.g., pharmacophobia), since any delays in prevention strategies would jeopardize public health.

One of the arguments most contested by conspiracy theorists is subordination of governments to “Big Pharma”. According to these theories, Big Pharma has led different governments to use a great number of economic resources on vaccine production, particularly those vaccines classified as “mRNA” vaccines, despite the fact that there are not scientific evidences on whether COVID-19 is a vaccine-eradicable disease or a stable over-time “vaccine-preventable disease” (17)in the context of:

(1) a globalized world (considering the high number of cases in low-income countries resulting in a high rate of viral mutations);

(2) a series of demographic projections that estimate a potential, future human overpopulation (which increases chance of the emergence and spread of epidemics and pandemics) probably associated with overconsumption (18, 19). Accordingly, it is interestingly to note that the COVID-19 pandemic is the first one that occurs with a human population of ~8 billion of people;

(3) a phase of the pandemic that is still under development.

The “mRNA vaccines” would be under experimentation for years, thus waiting for “the perfect storm” or a catastrophic infectious event to be tested on the market. Moreover, the new vaccines would be introduced on the market without adequate testing (the phases of the clinical research before postmarketing surveillance were performed in parallel rather than in series) and with a formal grant of use “for emergency only” by the main regulatory bodies. Of note, it has recently been proposed that RNA molecules may have an unexpected role in DNA preservation and regulation (20), although a virus generally involves the transfer of a greater amount of genetic material with respect to a simple vaccine.

In addition, a series of studies (e.g., clinical trials) based on potential reusable substances (e.g., hydroxychloroquine) led to their assessment of “lack of efficacy” or “excessive incidence of side effects” by several national regulatory authorities, so as to prevent these drugs from being formally approved for the treatment of COVID-19 (21, 22). This fact has been interpreted by conspiracy theorists as a boycott of therapies in favor of the diffusion of the vaccine.

Overall, the conspiracy theories regarding COVID-19 vaccines are generally fueled by three factors:

1. The uncertainty in communication was likely to have an important role. Specifically, it might be possible that information from the mass media is filtered in order to highlight an editorial point of view (e.g., some vaccines changed indications after news of specific adverse effects) (23). The same phenomenon would have happened in the scientific field in which several articles published in prestigious journals describing some drugs as ineffective has led to their underestimation in the treatment of COVID-19 (24). Accordingly, some of these papers would have been later retracted following warnings from different scientists (25).
2. The sales volume of some pharmaceutical companies dramatically increased due to the production of COVID-19 vaccines (26). According to the conspiracy theories, these profits would have been possible only under a monopoly regime. Moreover, the income imbalance between pharmaceutical companies and publishing houses would in turn have made the latter more susceptible to the influence of the former, thus facilitating the phenomenon of scientific misconduct (data fabrication/falsification) as occurred in the past (27, 28).
3. In some countries (e.g., Italy), governments would have implemented policies aimed at either requiring certain categories of people (e.g., health care workers) to get the vaccine (29) or to exclude those who had not accepted the vaccination from certain social situations (such as concerts, travel, or sporting events) (30).

The waiver of patent protections for COVID-19 vaccines may be useful for providing proof of efficacy for theorists of conspiracies, e.g., anti-vaccine (anti-vaxxers) groups. Indeed, after massive vaccination of the world's population, it will be possible to reduce COVID-19-related lethality and mortality, irrespective of the achievement of the herd immunity threshold.

## Negative Issues (Shadows)

On the other hand, the waiver of intellectual property protections on COVID-19 vaccines also implies a series of negative aspects that should be considered by low-income countries.

First, the resources materially necessary to produce vaccines should be carefully evaluated in future. Although present vaccines are not based on SARS-CoV-2 live virus strain amplification but on synthetic mRNA or genetic recombinant adenovirus vectors, the demand for new vaccines could increase in the coming years. Unfortunately, the tools and technologies required for this purpose are currently owned by only a few pharmaceutical companies. Moreover, not all countries would have on-site pharmaceutical industries that can guarantee adequate protective measures both for the technicians employed directly in the laboratories and for the population of the areas surrounding the laboratories themselves.

Clearly, a process involving the manipulation of viral strains is potentially dangerous for humans (for example, the accidental escape of potential pandemic viruses may determine future global epidemics), thus requiring high safety levels for the associated biological risk (31).

Second, health surveillance may not reach the same standards all over the world today. It should include the detection, assessment, understanding and prevention of vaccine adverse effects. This is a fundamental point since the first period of vaccination against COVID-19 in the West has publicly highlighted the possibility of some rare adverse effects that had not been reported in earlier stages of clinical research (phase 1, 2 and 3), in particular vaccine-induced thrombocytopenia/thrombosis associated with the use of viral vector vaccines (32–36). Unfortunately, national health surveillance procedures may not be standardized and their improvement depends on local laboratory and clinical capabilities (37).

Of note, weak health systems in low-income countries are likely to have fewer resources (e.g., feasible systems, governance, infrastructures, human resources, or skilled staff) to quickly intercept possible adverse events (38).

Third, potential problems concern the final product (the vaccine) that will be eventually developed in these centers (e.g., Africa or South America). Thus, production in conditions of imperfect biological quality control and lacking Good Manufacturing Practices (GMP) could be a harbinger of adverse reactions.

Fourth, the liberalization of vaccines might discourage pharmaceutical companies from investing in new vaccines or their updates in the future. In fact, the pandemic is still in an evolutionary phase, and more vaccines will most likely be needed in the coming years.

Furthermore, the success of vaccine sales and the underestimation of the usefulness of different drug therapies could lead some pharmaceutical companies to use their own production lines for vaccines at the expense of the production/development of experimental molecules.

Overall, all these problems are potentially associated with all vaccine types. Moreover, these problems can be amplified in times of pandemics and with greater access to vaccine production.

## Conclusion

Finally, from the point of view of the potential economic advantages, the decision to liberalize patents for COVID-19 vaccines has garnered the population's enthusiasm and various political exponents. However, from the point of view of safety and quality, this decision should be well considered. Moreover, vaccine production should be limited to facilities with adequate technical capacity and use of GMP to ensure product safety. Accordingly, this may require further financial investments and supportive technology transfer to low- and middle-income countries (LMICs). In fact, countries that, even now, manufacture a large fraction of the global vaccines, as India, could play a key role in ensuring access to COVID-19 vaccines for populations of low-income countries.

## Author Contributions

MN and PN: conceptualization, methodology, investigation, writing—original draft, and writing—review and editing. All authors contributed to the article and approved the submitted version.

## Conflict of Interest

The authors declare that the research was conducted in the absence of any commercial or financial relationships that could be construed as a potential conflict of interest.

## Publisher's Note

All claims expressed in this article are solely those of the authors and do not necessarily represent those of their affiliated organizations, or those of the publisher, the editors and the reviewers. Any product that may be evaluated in this article, or claim that may be made by its manufacturer, is not guaranteed or endorsed by the publisher.
